# Vestibular Migraine and Tinnitus: A Challenging Narrative

**DOI:** 10.7759/cureus.15998

**Published:** 2021-06-28

**Authors:** Alejandro García, Jorge Madrigal, Melissa Castillo-Bustamante

**Affiliations:** 1 Otolaryngology, Massachusetts Eye and Ear Infirmary, Boston, USA; 2 Otoneurology, Centro de Vértigo y Mareo, Mexico City, MEX

**Keywords:** vertigo diagnosis, migraine disorder, tinnitus, vestibular migraine, vestibular diseases

## Abstract

Vestibular migraine (VM) is one of the most common types of episodic vertigo. Over the last 11 years, this disorder has been studied by both neurologists and otolaryngologists. Based on the clinical symptoms and previous migraine history, the Barany Society and the International Classification of Headache Disorders have created the diagnostic criteria for VM. Unilateral and pulsatile headache, phonophobia, photophobia, auras, and a previous history of migraine have been included in these criteria. Although these symptoms are common and widely known, other symptoms not included in the Barany Society criteria are emerging and have been described in some clinical studies. These emerging criteria include audio-vestibular symptoms such as hearing loss, ear fullness, and tinnitus. Ringing ears could be associated with other vestibular disorders such as superior canal dehiscence and Ménière’s disease, but not in VM. The frequency, pathophysiological contributors, and clinical characteristics of this symptom in individuals with VM will be explored in this review.

## Introduction and background

Vestibular migraine (VM) is recognized as one of the most common causes of episodic vertigo, gaining increased recognition among neurologists, otolaryngologists, and neurotologists [1]. This condition has faced several modifications and diagnostic challenges in the last three decades [2]. It was first recognized in several case reports that some patients with migraines developed vestibular symptoms while others with undiagnosed episodic vestibular symptoms had migraine headaches.

In 2012, the Barany Society defined VM based on clinical criteria and recognized two different diagnoses: definite VM and probable VM (Table 1) [3]. The criteria were based on clinical symptoms including headache, vertigo, phonophobia, photophobia, and visual auras [3]. Although the presentation of these symptoms is still highly reported, during the last decade, other audiological symptoms such as tinnitus, hearing loss, and aural fullness have been described in VM patients [4,5]. Although these symptoms are commonly seen in other similar vestibular disorders including superior canal dehiscence syndrome and Ménière’s disease, in VM there is no permanent vestibular deficit or hearing loss [5]. We performed a narrative literature review to determine the frequency of tinnitus presentation in patients diagnosed with VM.

**Table 1:** Diagnostic criteria for definite vestibular migraine and probable vestibular migraine according to the Barany Society (2012). ICHD: International Classification of Headache Disorders

Definite vestibular migraine	
	A. At least five episodes with vestibular symptoms of moderate or severe intensity, lasting 5 minutes to 72 hours
	B. Current or previous history of migraine with or without aura according to the ICHD
	C. One or more migraine features with at least 50% of the vestibular episodes:
	– Headache with at least two of the following characteristics: one-sided location, pulsating quality, moderate or severe pain intensity, aggravation by routine physical activity
	– Photophobia and phonophobia
	– Visual aura
	D. Not better accounted for by another vestibular or ICHD diagnosis
Probable vestibular migraine	
	A. At least five episodes with vestibular symptoms of moderate or severe intensity, lasting 5 minutes to 72 hours
	B. Only one of the criteria B and C for vestibular migraine is fulfilled (migraine history or migraine features during the episode)
	C. Not better accounted for by another vestibular or ICHD diagnosis

## Review

Methodology

This review was conducted between March and April 2021. We used the MeSH headings “vertigo,” “migraine disorders,” and “tinnitus” to search PubMed, Embase, and Google Scholar databases for relevant articles. The search was limited to articles published between 2012, when the Barany Society published the VM diagnostic criteria, and 2021. We included complete manuscripts published in English and meeting the aims of the review. Studies outside the field of neurotology, neurology, and otolaryngology were excluded. Results were cross-checked among the three authors. In total, 103 indexed papers were identified in the initial search. From these, only 10 indexed articles were selected because they reported patients with VM and tinnitus. The quality of evidence in the published articles was reviewed according to the 2009 Levels of Evidence of the Oxford Centre for Evidence-Based Medicine.

Results

In the 10 studies (seven cross-sectional studies and three retrospective chart reviews), 951 patients with the diagnosis of VM not associated with other vestibular disorders reported tinnitus (Table 2). The estimated prevalence among the selected studies was 39.6% (range: 7-52.56%) [4-13]. According to one cross-sectional study, the average age for tinnitus presentation in VM patients was 38.6 years. No other studies presented similar data.

**Table 2:** Studies reporting tinnitus in patients with vestibular migraine.

Article	Country	Type of study	N	Tinnitus (%)
Yan et al. (2020) [6]	China	Cross-sectional	172	17-23
Van Ombergen et al. (2014) [4]	Belgium	Retrospective review	17	50
Teggi et al. (2017) [7]	Multiple countries	Cross-sectional	252	10.7
Radtke et al. (2011) [9]	Germany	Cross-sectional	61	10-33
Radtke et al. (2012) [8]	Germany	Cross-sectional	75	7-11
Power et al. (2018) [5]	Australia	Retrospective review	90	44
Morganti et al. (2016) [10]	Brazil	Cross-sectional	85	52.56
Neff et al. (2011) [11]	United States	Retrospective review	71	55
Kırkım et al. (2017) [11]	Turkey	Cross-sectional	44	36.4
Lopez-Escamez et al. (2014) [13]	Spain	Cross-sectional	84	20.2-26.2

Higher rates of VM were reported among females with an estimated prevalence of 64.7-84.9% [7-13]. Women in their sixth decade (mean: 50.5 years; range: 24-76) were more likely to report episodes of VM with concomitant tinnitus during and after vertigo attacks compared to males [4-12]. No previous noise exposure, surgical intervention, or acoustic trauma was reported in the selected studies [4-13]. One cross-sectional study reported the past medical history of patients and showed that there was a higher frequency of tinnitus in patients diagnosed with VM who reported a positive family history of migraine [7].

In terms of clinical presentation, patients with definite VM mostly presented with tinnitus compared to those with probable VM as seen in three cross-sectional studies [7,9,14]. Atypical VM due to lack of criteria for definite or probable VM was reported in one retrospective study revealing higher rates of tinnitus [4]. The onset of tinnitus observed in one cross-sectional study indicated diverse clinical appearance over the course of the disease [8]. During vertigo spells, tinnitus is less frequent than during the interval of attacks [8]. Frequent episodes of tinnitus more often present during the interval attack follow-up than after the acute episode [8]. However, Neff et al. reported nearly 38% of patients affected by tinnitus during vertigo spells [11]. According to the VM features and tinnitus onset, one study reported higher rates of tinnitus (23%) in patients who presented with earlier onset of migraine followed by those who presented with the concomitant onset of migraine and vertigo (22%) [6]. The description of side affected by tinnitus was described in two cross-sectional studies, showing that unilateral affection was more commonly reported in 54-68% of patients [12-14]. Both studies did not include patients with other vestibular disorders and concomitant tinnitus such as Ménière’s disease and superior canal dehiscence syndrome [12-14].

Other otologic symptoms were concomitantly reported in the studies. Among these symptoms, hearing loss and ear fullness were highly prevalent in VM patients. (Table 3) [4-11]. Although the main topic of this review is tinnitus, other otologic symptoms concomitant to tinnitus in VM episodes may be necessary to get an insight into the most common presentation.

**Table 3:** Other otologic symptoms in vestibular migraine. * Estimated prevalence during the interval of attacks; ** Prevalence obtained related to vertigo episodes.

Article	Type of study	N	Hearing loss (%)	Ear fullness (%)	Tinnitus (%)
Van Ombergen et al. (2014) [4]	Retrospective review	17	5	42	50
Teggi et al. (2017) [7]	Cross-sectional	252	4-15	8.8-30	10.7
Radtke et al. (2011) [9]	Cross-sectional	61	7-13	11-13	10-33
Radtke et al. (2012) [8]	Cross-sectional	75	12-26 15-38*	13-26 3-25*	7-11
Power et al. (2018) [5]	Retrospective review	90	23	30	44
Neff et al. (2011) [11]	Retrospective review	71	14-22 44**	27 70**	55
Lopez-Escamez et al. (2014) [13]	Cross-sectional	84	10.7-15.5	14.3-20.2	20.2-26.2

Discussion

About 38-46.4% of VM patients may experience tinnitus once during their lifetime [7-11]. VM patients with tinnitus present a higher prevalence than those without tinnitus (8-25%) [15]. Recognizing tinnitus associated with spontaneous and recurrent vertigo attacks may be difficult when distinguishing from other vestibular disorders [11,12]. Long VM attacks may mimic Ménière’s disease and short attacks are similar to benign paroxysmal positional vertigo (BPPV) episodes [10,11]. Tinnitus has been reported in both disorders making it difficult to precisely identify its origin [10].

Tinnitus in VM patients can be explained by a possible pathophysiologic hypothesis. This disorder presents central pathologic changes as vascular mechanisms, inflammatory responses, and neurochemical responses which lead to anatomic and functional changes within the inner ear and the central nervous system.

Vasospasm in small arterioles in the cochlea and labyrinth is widely known as a possible contributor of tinnitus onset in VM patients [14]. This may cause anatomic and volumetric changes within the endolymphatic sac, leading to endolymphatic hydrops [7,16]. This mechanism is similar to Ménière’s disease, making the onset of tinnitus indistinguishable when both diseases overlap or one of them is underdiagnosed [17]. One possible difference between both entities is the increased prevalence of tinnitus in patients with Ménière’s disease compared to VM (49% vs 38%) [17]. Another controversial fact is the lower female-to-male ratio (1:1.3) in patients with Ménière’s disease and the age of presentation between the fourth to seventh decades of life [17]. In the audiologic setting, hearing loss in Ménière’s disease is usually progressive [17]. Nonetheless, further studies are needed to understand the precise pathophysiological events related to tinnitus presentation in VM.

Another challenge differentiating tinnitus associated with VM is the presence of migraine attacks where VM is not the primary culprit, which is the case among patients with BPPV and migraine with brainstem aura [18]. Parker reported a strong association between migraine and vertigo explained by defective calcium channels primarily expressed in the inner ear and cerebral cortex, which could lead to reversible hair cell depolarization and posterior auditory and vestibular symptoms [19]. This has been reported by clinical and audiological assessments where patients with migraines present with tinnitus and hearing abnormalities. Some of these findings are shown in abnormal pure-tone average as well as auditory brainstem response changes in wave I demonstrated by prolonged absolute latency [20]. Even though these findings are not reported in VM, they should be considered when differentiating tinnitus associated with a migraine from those related to VM.

To our knowledge, this is one of the first reports summarizing the incidence of tinnitus in VM. Although cross-sectional and retrospective studies revealed a variable incidence and presentation of tinnitus in patients with VM without other vestibular conditions, further extensive clinical studies should be conducted to characterize this symptom during migraine attacks and the interval of symptomatic episodes. The increased incidence of tinnitus could be an indicator of cochlear and/or auditory nerve involvement in patients with VM [12]; however, objective audiometric and electrophysiological data are still necessary during migraine attacks. The main limitations of this narrative review are the small number of studies included and the need to include other relevant characteristics such as duration, intensity, and clinical questionnaires to describe tinnitus in patients with VM.

## Conclusions

In this literature review, an increased prevalence of unilateral tinnitus with a variable presentation during vertigo attacks in VM is reported. Recognizing tinnitus may be challenging when differentiating from other vestibular and central nervous system disorders. All vestibular symptoms associated with migraines are transient. Further clinical studies are needed to understand the real involvement of tinnitus in VM.
